# Systematic Review of Traumatic Brain Injuries in Baseball and Softball: A Framework for Prevention

**DOI:** 10.3389/fneur.2017.00492

**Published:** 2017-10-30

**Authors:** Michael D. Cusimano, Alice Zhu

**Affiliations:** ^1^Division of Neurosurgery, Department of Surgery, St. Michael’s Hospital, Injury Prevention Research Office, Li Ka Shing Knowledge Institute, Keenan Research Centre, University of Toronto, Toronto, ON, Canada; ^2^Dalla Lana School of Public Health, University of Toronto, Toronto, ON, Canada

**Keywords:** traumatic brain injury, baseball, softball, concussion, head injury, brain injury, prevention

## Abstract

**Background:**

Traumatic brain injuries (TBIs) are an important public health challenge. The classification of baseball and softball as low contact sports and their association with extremity injuries cause individuals to overlook the risk of TBI in baseball and softball.

**Purpose:**

To summarize our knowledge of the epidemiology and risk factors of TBIs associated with baseball and softball with an aim to better design and implement preventive strategies.

**Methods:**

A search algorithm containing keywords that were synonymous to the terms “TBI,” “baseball” was applied to the following nine databases: MEDLINE, Scopus, PubMed, EMBASE, CINAHL, Healthstar, PsychINFO, AMED, Cochrane library. Cited reference lists of identified articles were also consulted yielding a total of eighty-eight articles for full review. The search was concluded on November 14, 2016. The level of evidence was evaluated according to the guidelines from Strengthening the Reporting of Observational Studies in Epidemiology statement.

**Results:**

Twenty-nine articles published between 2000 and 2016 met the criteria for analysis. Collectively, they examined the years 1982–2015 and identified 242,731 baseball-and softball-related TBIs. The most explored outcome of TBI was concussion. The average injury rate per 1,000 athletic exposures was 0.13 (range 0.03–0.46). The most common mechanism of injury was being struck by bat for younger players and being struck by ball for older athletes (adolescent and beyond). Rates of TBI were on average 4.17 times greater in games compared to practices. Females were on average 2.04 times more likely to sustain a TBI than males. Severity of TBIs varied considerably from mild and returning to the field on the same day, to immediate death. Generally, there is poor compliance with helmet use and return-to-play post-concussion guidelines. An increase TBI rates was observed over time. Multifaceted preventive strategies must be implemented to reduce the frequency and burden of these injuries.

**Conclusion:**

It is difficult to compare the epidemiologic trends of TBI in baseball and softball due critical differences in the methods employed across the studies. Additional research is needed to provide a greater understanding of baseball- and softball-related TBI and to aid in the development of prevention and management modules.

## Introduction

Each year, over six million children play in organized baseball leagues and up to thirteen million more play non-organized baseball (1). Given that baseball/softball is most commonly played in North America, most literature to date comes from North America, with very little arising from Central America, Asia, the Caribbean, and other countries. Most injuries in baseball and softball involve the upper and lower extremities (2, 3). However, with balls being thrown at high speeds (4, 5), the use of bats and the potential for player to player collision, it is not surprising that baseball and softball players can suffer potentially serious injuries to the head and brain. The burden of head trauma related to baseball and softball requires further investigation. For example, in 2009, 38,942 patients with baseball-related head injuries were treated in American emergency rooms alone. However, many more individuals suffered head injuries but did not seek care in an emergency department (ED) (6). Previous papers have studied these injuries, but only as parts of larger reviews of TBIs across multiple sports and as such the amount of information is limited (7, 8). Given the incidence of head injuries and the potentially lifelong effects of TBIs, it is imperative to undertake steps directed toward the prevention of baseball-and softball-related TBIs. Before effective strategies can be implemented and evaluated, the factors associated with baseball and softball TBI must be well understood. The purpose of our study is to summarize the current published literature related to TBIs in baseball and softball, to identify avenues for injury prevention and to identify gaps in the literature giving potential insight into future research.

## Methods

### Protocol

Our methodology and reporting has been done according to the Preferred Reporting Items for Systematic Reviews and Meta-analysis (PRISMA) guidelines (9). PRISMA is evidence based minimum checklist for reporting in systematic reviews and meta-analysis. PRISMA provides authors with a framework and ensures for high quality reporting in systematic reviews. It is recommended by the International Committee of Medical Journal Editors and is referred to in the Uniform Requirements for Manuscripts Submitted to Biomedical Journals (10).

### Article Selection Criteria

**Figure 1:**
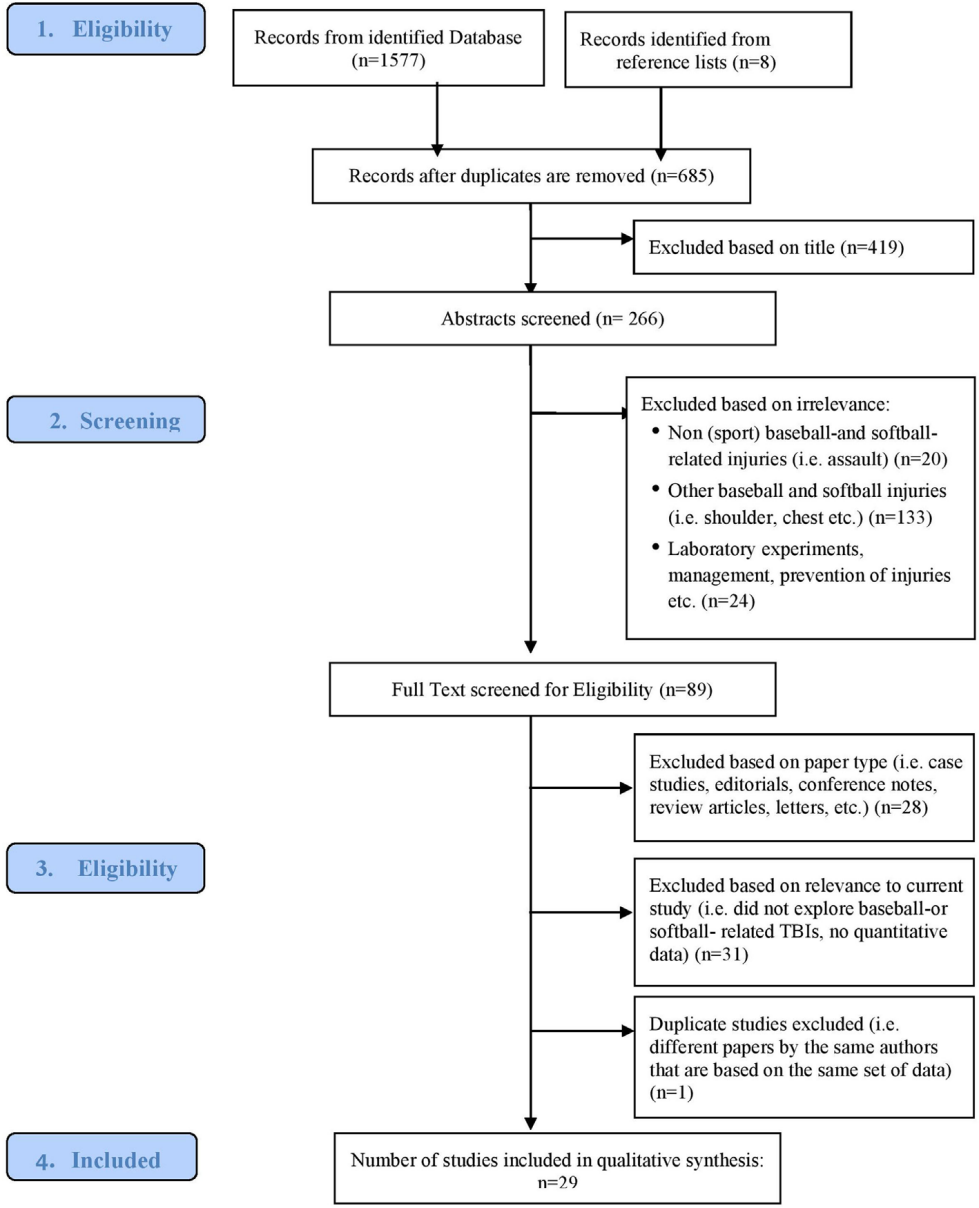
Consort diagram.

Identified studies were manually reviewed for relevance through the application of inclusion and exclusion criteria (Figure 1). Peer-reviewed literature that focused on TBI or concussion in baseball, softball, or T-ball was included. All the following were excluded (1) studies that do not have samples that included baseball, softball, or T-ball players; (2) studies solely examining non-TBI alone, such as only ocular, facial, or cardiac and extremities injuries; (3) original research studies that were not published in peer-reviewed journals; (4) review articles; (5) abstracts, comments, case reports, newspaper articles, and conference notes; (6) laboratory studies on prevention methods (e.g., testing hardness of various ball types *via* Hybrid III dummy).

### Information Sources

Using search terms including: “Traumatic brain injury,” “TBI,” “craniocerebral trauma,” “concussion,” “cerebral contusion,” “head injury,” “hemorrhage,” “hematoma,” “brain damage,” “mild traumatic brain injury,” “mTBI,” “penetrating brain,” “closed head injury,” “baseball,” “softball,” “MLB,” “major league baseball,” “minor league baseball,”” MiLB,” “tee ball,” and “t-ball,” all published articles related to TBI and baseball, softball, and T-ball were reviewed through Medline, EMBASE, CINAHL, PubMed, Healthstar, PsychINFO, AMED, Cochrane Library, and Scopus. Cited reference lists of identified articles were also consulted. The search concluded on November 14, 2016. Our Medline search strategy for articles from 1946 to November Week 1 2016 is presented in Table 1.

**Table 1:** Search results from Ovid MEDLINE.

Database: Ovid MEDLINE(R) <1946 to November Week 1 2016> Search Strategy:
1. brain concussion.mp. or exp brain concussion/(6,556)
2. brain injury.mp. or exp brain injury/(78,172)
3. concuss$.mp. (8,157)
4. traumatic brain injur$.mp. (24,557)
5. TBI.mp. (17,201)
6. craniocerebral trauma.mp. or exp craniocerebral trauma/(145,610)
7. head injur$.mp. (23,582)
8. [(head adj6 baseball) or (concuss$ adj6 baseball)].mp. (43)
9. exp “wounds and injuries”/and (head or head$).mp. (56,228)
10. exp hemorrhage/or exp hematoma/or blood clots.mp. (301,936)
11. cerebral contusion.mp. (468)
12. (penetrating adj6 brain).mp. (921)
13. [closed head injur$ or (brain adj6 damage)].mp. (37,648)
14. (minor head injur$ or minor traumatic brain injur$ or MTBI).mp. (2,162)
15. 1 or 2 or 3 or 4 or 5 or 6 or 7 or 8 or 9 or 10 or 11 or 12 or 13 or 14 (501,336)
16. exp baseball/or baseball.mp. (2,807)
17. exp softball/or softball.mp. (2,298)
18. catcher.mp. (209)
19. ump$.mp. (2,112)
20. pitcher.mp. (463)
21. baseman.mp. (17)
22. short stop.mp. (53)
23. outfielder.mp. (6)
24. foul ball.mp. (3)
25. bunt.mp. (93)
26. (spectator adj6 baseball).mp. (3)
27. (MLB or major league baseball or minor league baseball or MiLB).mp. (393)
28. (tee ball or t-ball or tball).mp. (5)
29. infielder.mp. (6)
30. 16 or 17 or 18 or 19 or 20 or 21 or 22 or 23 or 24 or 25 or 26 or 27 or 28 or 29 (5,793)
31. 15 and 30 (292)
32. remove duplicates from 31 (269)

### Data Extraction

Data from the included studies were extracted twice independently using a standardized and piloted electronic form (Microsoft Excel). Any differences in extraction were resolved by consensus. Information was extracted on the publication (first author name, publication year), study details (country of origin, design, injury reporting system used, length of study, setting), TBI definition, sample size, use of protective equipment and outcomes (injury rate injury severity), and mechanism of injury. Studies were also categorized to MLB (a professional baseball organization in the United States and Canada with 30 teams and 750 active players), Minor League Baseball (network of clubs that are each associated with a major league club; consisting of approximately 7,500 players), Little League Baseball (organization for young baseball players ages 7–18), high school, university/collegiate, and by age groups (players ages 0–19 are considered as youth).

### Summary Measures

Figure 1 presents the consort diagram and the characteristics and overview of included studies are illustrated in a summary table. Specific definition of TBI, mechanism of injury, use of protective equipment, gender differences, post injury outcomes, TBI rates, and any additional trends the study observed were recorded and are also summarized in tables.

### Methodological Quality of Studies

The Strengthening the Reporting of Observational Studies in Epidemiology (STROBE) statement (2008) was used to evaluate the overall strength of all 29 included studies (11). The checklist was designed to measure the methodological quality of observational studies, including cohort, case–control, and cross-sectional studies. Its use is endorsed by a growing number of biomedical journals and is recommended by the International Committee of Medical Journal Editors (10).

The checklist consists of 22 criteria resulting in a maximum of 22 points, with higher scores corresponding to superior qualities. Articles with quality scores from 16–22 were considered “good,” 9–15 as “moderate” and less than 9 as “poor.” Articles were assessed by two research assistants and given scores were averaged. The raters did not differ by more than three points in their quality assessment of any single study, and different raters’ scores were averaged.

## Results

Characteristics of the included studies and definitions of TBI are summarized in Tables 2 and 3. Rates of injury, mechanisms of injury and protective equipment wear, and injury outcomes are summarized in Tables 4–6, respectively. Table 7 provides a framework to conceptualize, predict, prevent, and mitigate baseball- and softball-related TBIs.

**Table 2:** Characteristics of included studies.

Reference	Country (year)	Design (period of study)	Record	Age	# TBI (%^a^)	Setting (formal vs. recreational league)	Injury type	Strobe
Athiviraham et al. (4)	USA (2012)	Retrospective case-control (2009 and 2010 seasons)	Video coverage MLB	NR	9 (NR)	MLB (Formal)	Concussion	17
Bakhos et al. (12)	USA (2010)	Retrospective review of databases (NEISS 1997–2007; NEISS-AIP 2001–2005)	NEISS	8–19	6,872^b^ (NR)	ED of 98 Hospitals across the US	Concussion	19
Beyer et al. (5)	USA (2012)	Retrospective case-control (2008, 2009, 2010 seasons)	Video coverage MLB	NR	10 NR)	MLB-catchers and umpires with helmet (Formal)	Concussion	19
Birchak et al. (13)	USA (2013)	Retrospective cohort study (1994–2010)	NEISS	7–96	126,469 (6.0%)	ED of 98 Hospitals across the US	Head injury	22
Boden et al. (2)	USA (2004)	Retrospective cohort study (1982–2002)^c^	NCCSIR	13–40	26 (NR)	US high schools and colleges (organized recreational)	Head injury	16
Cheng et al. (14)	USA (2000)	Retrospective cohort study (1996–1998)^c^	District of Columbia Adolescent ISS	10–19	37 (NR)	ED of 7 US Regional hospital trauma center	Head injury	20
Collins and Comstock (3)	USA (2008)	Retrospective comprehensive study (2005–2007)	Reporting Information Online (RIO)	NR	15 (3.5%)	100 US high schools (organized recreational)	Concussion	22
Covassin et al. (15)	USA (2003)	Prospective population based Injury Surveillance (1997–2000)	NCAA ISS	NR	159^b^ (4.5%)	US colleges (Organized recreational)	Concussion	20
Covassin et al. (16)	USA (2016)	Descriptive epidemiologic study (2004/2005–2008/2009)	NCAA ISS	NR	173^b^ (NR)	US colleges (Organized recreational)	Concussion	19
Cusimano et al. (17)	Canada (2013)	Retrospective case series (1990–2009)	CHIRPP	5–19	835^b^ (NR)	ED of 11 pediatric and 4 general Canadian hospitals	Brain Injury	21
Dick et al. (18)	USA (2007)	Retrospective cohort study (1988/1989–2003/2004)^c^	NCAA ISS	NR	210 (2.5%)	US Colleges: Division I, II, and III (organized recreational)	Concussion	9
Gessel et al. (19)	USA (2007)	Descriptive epidemiologic study (2005–2006)	Reporting Information Online (RIO); NCAA ISS	NR	46^b^ (4.2%)	US High schools and colleges (organized recreational)	Concussion	21
Green et al. (20)	USA (2015)	Descriptive epidemiologic study (2011–2012)	MLB Electronic Medical Record and Health and Injury Tracking System	NR	307 (NR)	Major and minor baseball leagues in the US	MTBI (concussion)	22
Hanson et al. (21)	USA (2013)	Retrospective study (2002–2011)	Hospital based ISS: Cincinnati Children’s Hospital Medical Center	0–19	344^b^ (NR)	ED at an US level 1 trauma center	TBI	21
Hootman et al. (22)	USA (2007)	Retrospective cohort study (1988/1989–2003/2004)	NCAA ISS	NR	438^b^ (3.2%)	US colleges: Division I, II, III (Organized recreational)^b^	Concussion	16
Kerr et al. (23)	USA (2016)	Descriptive epidemiologic study (2011/2012–2014/2015)	NCAA ISS	NR	51^b^ (NR)	US colleges (Organized recreational)	Concussion	21
King et al. (24)	New Zealand (2014)	Retrospective case review (2001–2011)	Accidence Compensation Corporation claims & costs	NR	27^b^	Insurance claims	Concussion	19
Lawson et al. (25)	USA (2009)	Retrospective cohort study (1994–2006)^c^	NEISS	0–17	91,877 (5.8%)	ED of 98 Hospitals across the US	Concussion/CHI	21
Lincoln et al. (26)	USA (2011)	Prospective: descriptive epidemiology study (97–08)	District-mandated electronic medical record	NR	79^b^ (NR)	25 High schools in one US public school system (organized recreational)	Concussion	20
Marar et al. (27)	USA (2012)	Descriptive epidemiologic study (2008–2010)	Reporting Information Online (RIO)	NR	81^b^ (NR)	100 US High schools (organized recreational)	Concussion	20
Marshall et al. (28)	USA (2007)	Prospective population based Injury Surveillance (1988/89–2003/04 Seasons)^c^	NCAA ISS	NR	228 (4.3%)	US Colleges: Division I, II, III (organized recreational)	Concussion	11
Mueller (29)	USA (2001)	Retrospective cohort study (1982–1999)^c^	NCCSIR, NCAA, National Federation of state High School Associations	NR	17 (NR)	US High schools and colleges (organized recreational)	Catastrophic HI	15
Pasternack et al. (30)	USA (1996)	Prospective population based injury survey (1994 Season)^c^	League based, managers’ report to the survey	7–18	3 (3.7%)	An US Little League Baseball (Formal)	Head injury	18
Powell (31)	USA (1999)	Prospective cohort study (1995–1997 Academic years)	NATA ISS	NR	40^b^ (2.2%)	US High schools (organized recreational)	MTBI	20
Rechel et al. (32)	USA (2008)	Prospective injury surveillance study (2005–2006 Academic years)^c^	Reporting Information Online (RIO)	NR	4,153 (3.2%)	100 US High Schools (organized recreational)	Concussion	20
Rosenthal et al. (33)	USA (2014)	Descriptive epidemiologic study (2005/2006–2011/2012)	Reporting Information Online (RIO)	NR	46^b^ (NR)	100 US High schools (organized recreational)	Concussion	21
Wasserman et al. (34)	USA (2015)	Retrospective cohort study (2007–2013)	MLB disabled-list records; a Baseball Prospectus	NR	66 (NR)	MLB (Formal)	Concussion	19
Wasserman et al. (35)	USA (2016)	Descriptive epidemiologic study (2009/2010–2013/2014)	NCAA ISS	NR	69^b^ (NR)	US colleges (Organized recreational)	Concussion	21
Yard and Comstock (36)	USA (2009)	Prospective cohort study (2005–2008)	Reporting Information Online (RIO)	NR	NR	100 US High schools (organized recreational)	Concussion	21

*MLB, Major League Baseball; NEISS, National Electronic Injury Surveillance System; NATA, National Athletic Trainer Association; ISS, injury Surveillance system; NCAA, National Collegiate Athletic Association; NCCSIR, National Center for Catastrophic Sports Injury Research; CHIRPP, Canadian Hospitals Injury Reporting and Prevention program; NR, data not reported; ED, emergency department; ATC, Certified Athletic Trainer; MTBI, Mild Traumatic Injury; CHI, Closed Head Injury; SHI, Severe head injury*.

*^a^% TBI of total baseball-related injuries*.

*^b^Study also observed other sports, only those relevant to study were included*.

*^c^Study also observed other baseball-related injuries, only injuries relevant to study were included*.

**Table 3:** Definition and diagnosis of TBI.

Reference	Injury type	Definition of concussion/TBI	Diagnosis source
Athiviraham et al. (4)	Concussion	Not specified	Team physician
Bakhos et al. (12)	Concussion	Not specified	ED physician
Beyer et al. (5)	Concussion	Not specified	Clinical diagnosis of concussion or reported concussive symptoms
Birchak et al. (13)	Head injury	Injury to anatomical head (include but not limited to: concussions and closed head injuries)	ED physician
Boden et al. (2)	Head injury	Not specified	Reports to the NCCSIR from high school and college coaches, athletic trainers, athletic directors, executive offices of state, national athletic organizations and a national newspaper clipping service
Cheng et al. (14)	Head injury	Injury to anatomical head (include but not limited to: open head wound and closed head injuries)	ED physician
Collins and Comstock (3)	Concussion	Not specified	Team certified athletic trainer or physician
Covassin et al. (15)	Concussion	NCAA concussion scale	Team certified athletic trainer or physician
Covassin et al. (16)	Concussion	NCAA concussion scale	Team certified athletic trainer or physician
Cusimano et al. (17)	Brain injury	Include but not limited to: minor closed head injury, concussion (not specified), and intracranial injury	ED physician
Dick et al. (18)	Concussion	NCAA concussion scale	Team certified athletic trainer and/or physician
Gessel et al. (19)	Concussion	NCAA concussion scale	Team certified athletic trainer and/or physician
Green et al. (20)	MTBI	Complex pathophysiological process affecting the brain, induced by biomechanical forces diagnosed by a physician	Team physician and/or ED physician
Hanson et al. (21)	TBI	ICD-9 code (800.0–801.9, 803.0–804.9, 850.0–854.1, 950.1–950.3, 995.55, and 959.01	ED physician
Hootman et al. (22)	Concussion	NCAA concussion scale	Team certified athletic trainer and/or physician
Kerr et al. (23)	Concussion	NCAA concussion scale	Team certified athletic trainer and/or physician
King et al. (24)	Concussion	Accident Compensation Corporation code S60	Moderate to serious insurance claims
Lawson et al. (25)	Concussion/CHI	Not specified	ED physician
Lincoln et al. (26)	Concussion	Standardized Assessment of Concussion scores (CSMi, Medical Solutions, Stoughton, CA, USA)	Certified athletic trainer
Marar et al. (27)	Concussion	Not specified	Team certified athletic trainer and/or physician
Marshall et al. (28)	Concussion	NCAA concussion scale	Team certified athletic trainer and/or physician
Mueller (29)	Catastrophic head injury	Severe injury to head (Include but not limited to: subdural hematoma, brain injury, aneurysm)	Reports to the NCCSIR from high school and college coaches, athletic trainers, athletic directors, executive offices of state, national athletic organizations and a national newspaper clipping service
Pasternack et al. (30)	Head injury	Injury to anatomical head	Team manager and/or physician
Powell (31)	MTBI	Players removed from participation to be evaluated (by athletic trainer and/or physician) for traumatic brain or head injury prior to returning to play	Team certified athletic trainer and/or physician
Rechel et al. (32)	Concussion	Not specified	Team certified athletic trainer and/or physician
Rosenthal et al. (33)	Concussion	Not specified	Team certified athletic trainer and/or physician
Wasserman et al. (34)	Concussion	Not specified	Not specified
Wasserman et al. (35)	Concussion	NCAA concussion scale	Team certified athletic trainer and/or physician
Yard and Comstock (36)	Concussion	AAN concussion and Prague concussion guidelines	Team certified athletic trainer

*NCAA concussion scale: Injury must have restricted participation for one or more days beyond day of injury. Grade 1: No LOC, short duration of post-traumatic amnesia; Grade 2: LOC < 5 min amnesia up to 30 min Grade 3: LOC > 5 min extended amnesia*.

*AAN concussion scale: Grade 1: transient confusion, no LOC, symptoms < 15 min. Grade 2: Transient confusion, no LOC, symptoms > 15 min. Grade 3: any LOC*.

*Prague: Simple: symptoms resolve within 7–10 days. Complex: symptoms are persistent, LOC > 1 min, prolonged cognitive impairment, athlete has suffered multiple concussions*.

**Table 4:** Rates of baseball-and softball-related TBI.

Reference	Setting/participants	Definition of TBI	Definition of rate	Population	TBI injury rate			IRR games: practice^a^	IRR women: men^^b^^
					Total	Games	Practices		
Bakhos et al. (12)	Emergency department	Concussion	TBI per 10,000 participants	Ages 7–11	1	NR	NR	N/A	N/A
				Ages 12–17	3	NR	NR	N/A	N/A
Covassin et al. (15)	College athletes	Concussion	TBI per 1,000 AE	Men’s baseball	NR	0.29	0.06	3.80	N/A
				Women’s softball	NR	0.31	0.13	2.50	
Covassin et al. (16)	College athletes	Concussion	TBI per 1,000 AE^c^	Men’s baseball	0.12^c^	0.26^c^	0.05^c^	5.20	1.95
				Women’s softball	0.23^c^	0.37^c^	0.14^c^	2.64	
Dick et al. (18)	College athletes	Concussion	TBI per 1,000 AE	Men’s baseball	NR	0.19	0.03	6.33^e^	N/A
Gessel et al. (19)	High school athletes	Concussion	TBI per 1,000 AE	Men’s baseball	0.05	0.08	0.03	2.67^e^	1.48
				Women’s softball	0.07	0.04	0.09	0.44^e^	
	College athletes	Concussion	TBI per 1,000 AE	Men’s baseball	0.09	0.23	0.03	7.67^e^	2.11^e^
				Women’s softball	0.19	0.37	0.07	5.29^e^	
Green et al. (20)	Major and minor league	MTBI	TBI per 1,000 AE	Major league	0.26	NR	NR	N/A	N/A
				Minor league	0.46	NR	NR	N/A	N/A
Hootman et al. (22)	College athletes	Concussion	TBI per 1,000 AE	Men’s baseball	0.07	NR	NR	N/A	2.00^e^
				Women’s softball	0.14	NR	NR	N/A	
Kerr et al. (23)	College athletes	Concussion	TBI per 1,000 AE	Men’s baseball	0.09	0.16	0.04	4.00^e^	3.01
				Women’s softball	0.26	0.42	0.18	2.33^e^	
King et al. (24)	Insurance claims	Concussion	TBI per 1,000 claim	Men	0.90	NR	NR	N/A	0.44^e^
				Women	0.40	NR	NR	N/A	
Lincoln et al. (26)	High school athletes	Concussion	TBI per 1,000 AE	Men’s baseball	0.06	NR	NR	N/A	1.83^e^
				Women’s softball	0.11	NR	NR	N/A	
Marar et al. (27)	High school athletes	Concussion	TBI per 1,000 AE^c^	Men’s baseball	0.05^c^	0.11^c^	0.01^c^	11.00	3.20
				Women’s softball	0.16^c^	0.29^c^	0.09^c^	3.22	
Marshall et al. (28)	College athletes	Concussion	TBI per 1,000 AE	Women’s softball	NR	0.25	0.07	3.57^e^	N/A
Powell (31)	High school athletes	MTBI	TBI per 1,000 AE	Men’s baseball	0.05	0.12	0.03	4.50	2.00^e^
				Women’s softball	0.10	0.13	0.08	1.63^e^	
Rosenthal et al. (33)	High school athletes	Concussion	TBI per 1,000 AE	Men’s baseball	0.03	NR	NR	N/A	2.33^e^
				Women’s softball	0.07	NR	NR	N/A	
Yard and Comstock (36)	High school athletes	Concussion	TBI per 1,000 AE^d^	Men’s baseball	0.03^d^	NR	NR	N/A	2.06^e^
				Women’s softball	0.07^d^	NR	NR	N/A	

*AE, athletic exposure; NR, data was not reported*.

*^a^Injury Rate Ratio (IRR) obtained *via* dividing the injury rate of games by rate of practices*.

*^b^IRR obtained via dividing the women’s total injury rate of by men’s total injury rate*.

*^c^Rate was changed from 10,000 AE (originally presented in paper) to 1,000 AE in order to make the data comparable*.

*^d^Rate was changed from 100,000 AE (originally presented in paper) to 1,000 AE in order to make the data comparable*.

*^e^Rate ratio extracted from given rates*.

**Table 5:** Mechanisms of TBI and protective equipment wear.

Reference	Total TBI (*n*)	Investigated mechanism	*n* (%)^a^	With protective equipment *n* (%)^a^	
Athiviraham et al. (4)	9	Hit by ball (pitch)	9 (100%)	Helmet	9 (100%)

Beyer et al. (5)	10	Hit by ball (batted)	10 (100%)	Helmet	10 (100%)

Birchak et al. (13)	126,469^b^	Hit by ball (batted)	95,737^b^ (75.7%)	NR	

Boden et al. (2)	26	Collision between players	9 (34.6%)	Helmet	0 (0%)
		Hit by ball (batted)	15 (57.7%)		
		Hit by ball (pitch)	2 (7.7%)		

Cheng et al. (14)	37	Hit by ball	11 (29.7%)	NR	
		Hit by bat	5 (13.5%)		
		Not reported	21 (56.8%)		

Collins and Comstock (3)	15	Hit by ball	4 (8%)	Helmet	Poor (N/A)
		Others	11 (73.3%)		

Cusimano et al. (17)	835	Collision between players	79 (9.5%)	Helmet	56 (6.7%)
		Hit by ball/bat	699 (83.7%)	Others	54 (6.5%)
		Struck surface	10 (1.2%)	None	106 (12.7%)
		Others	47 (5.6%)	Unspecified	619 (74.1%)

Dick et al. (18)	210	Hit by ball (batted-during games)	89 (42.4%)	NR	

Gessel et al. (19)	5,549^c^	Hit by ball	3,922^c^ (70.7%)	NR	

Green et al. (20)	307	Collision between players	91 (29.6%)	NR	
		Hit by ball (batted)	61 (19.9%)		
		Hit by ball (non-pitch)	23 (7.5%)		
		Hit by ball (pitch)	79 (25.7%)		
		Hit by bat	12 (3.9%)		
		Others	41 (13.4)		

Marar et al. (27)	23	M baseball: hit by ball (pitch)	6 (26.1%)	NR	
		M baseball: hit by ball (batted)	6 (26.1%)		
	58	F softball: hit by ball (pitch)	3 (5.2%)	NR	
		F softball: hit by ball (batted)	16 (27.6%)		

Marshall et al. (28)	151	Hit by ball (batted-during games)	75 (49.7%)	NR	

Powell (31)	40	Collision between players	22 (55.0%)	NR	
		Hit by bat	4 (10.0%)		
		Hit by ball (batted)	2 (5.0%)		
		Hit by ball (pitch)	5 (12.5%)		
		Sliding	7 (17.5%)		

Total	133,739	Struck by implement	100,773 (75.4%)	N/A	
		Hit by ball	100,053 (99.3%)		
		Hit by bat	21 (0.02%)		
		Unspecified (ball/bat)	699 (0.7%)		
		Collision between players	196 (0.15%)		
		Sliding	12 (0.01%)		
		Others/unspecified	32,686 (24.5%)		

*^a^Frequency and percentages are based on all the known cases of TBI identified in the respective study*.

*^b^Estimated number by extrapolation*.

*^c^National estimates reported by authors*.

**Table 6:** Injury outcomes.

Reference	Injury type	Injury outcomes/severity	
Athiviraham et al. (4)	Concussion	Avg. days missed Players observed LOC	14.2 days (range 1–22) *n* = 0

Boden et al. (2)	Concussion	Death	*n* = 5 (21.7%)
		Survivors	*n* = 18 (78.3%)
		Survivors requiring ≥ 1 surgical procedure	*n* = 8 (34.8%)
		Survivors with post injury neurological symptoms	*n* = 11 (47.8%)
		Survivors with full recovery	*n* = 5 (21.7%)

Covassin et al. (16)	Concussion	Avg. days missed from practice concussions	Baseball 7.56 daysSoftball 7.97 days
		Avg. days missed from competition concussions	Baseball 8.04 daysSoftball 8.19 days
Gessel et al. (19)	Concussion	Players with symptoms resolved within 6 days	Baseball *n* = 1,279 (64.2%)
			Softball *n* = 2,449 (68.8%)

Green et al. (20)	MTBI	Median days missed	9 days

Lawson et al. (25)	Concussion/CHI	Players requiring hospital admission after ED visit	*n* = 4,317

Mueller (29)	Head injury	Death	*n* = 3 (19%)
		Survivors with post injury disability	*n* = 5 (31%)
		Survivors with full recovery	*n* = 8 (50%)

Pasternack et al. (30)	Head injury	Players returning to play < 1 month post injury	*n* = 3 (100%)

Powell (31)	MTBI	Players returning to play < 8 days	Baseball *n* = 8 (53.3%)
			Softball *n* = 23 (88.5%)
		Players returning to play 8–21 days	Baseball *n* = 7 (46.7%)
			Softball *n* = 3 (11.5%)
		Median days missed	Baseball 3 days
			Softball 2 days

Wasserman et al. (34)	Concussion	Players returning to play < 6 days	*n* = 21 (31.8%)
		Players returning to play < 10 days	*n* = 38 (57.6%)
		Players returning to play < 30 days	*n* = 62 (93.9%)
		Players missed > 30 days	*n* = 4 (6.1%)

Wasserman et al. (35)	Concussion	Avg. Number of symptoms of concussion	Baseball 4.69 Softball 5.96
		% Players with symptoms lasting > 4 weeks	Baseball 1.9% Softball 18.8%
		% Players reported having headache post concussion	Baseball 75% Softball 94.2%

Yard and Comstock (36)	Concussion	% Of concussion rated AAN Grade I	Baseball 11.1% Softball 17.8%
		% Of Concussion rated AAN Grade II	Baseball 22.3% Softball 75.0%
		% Of Concussion rated AAN Grade III	Baseball 66.6% Softball 7.1%

**Table 7:** Haddon’s Matrix for prevention initiatives.

Phases	Host	Agent	Physical environment	Social/economic environment
Pre-injury	– Velocity of pitch – Attitude of athlete (aggressive, competitive) – Athlete age and sex – Athlete strength	– Hardness/density of ball and bat – Inadequate protective gear – Design and type of helmet	– Maintenance of the field/grounds – Weather/time of year Formal/informal setting	– Public perception of wearing protective gear – Costs of protective gear

Injury	– Unaware of the potential dangers of equipment (i.e., bat) – Lack of supervision of younger athletes – Lack of education to kids	– Hardness of the ball/bat – Association of bat and ball exit velocity	– Surface hardness – Obstacles on field – Personal protective equipment	– Enforcement of rules and laws (ZZL) – Enforcement of protective gear use

Post-injury	– Knowledge to report symptoms – Compliance with return-to-play guidelines	– Engineering-improved helmet, bat and ball design	– Access to a hospital or trauma center	– Expense/cost of medical system – Evaluation of surveillance systems – Insurance rates, fines – Social support – Community response to TBI

### Study Selection

Published between 1996 and 2016, the 29 studies collectively examined the years 1982–2016 (Table 2). Of the included studies, 23 were published after 2005, illustrating the increase of TBI research in recent years. Geographically, studies covered the United States (*n* = 27), Canada (*n* = 1), and New Zealand (*n* = 1). A variety of sources for TBIs in baseball and softball were identified including hospital or trauma center’s ED (*n* = 6), national and district injury surveillance/reporting systems (*n* = 17), MLB video coverage (*n* = 2), MLB electronic medical records or disabled list (*n* = 2), independent researchers (*n* = 1), and insurance claims (*n* = 1). Comprehensive data were also gathered from high school and collegiate level reporting systems using certified athletic trainers. Although each source provided a limited view of baseball- and softball-related TBIs, together they provided practical insight and allowed for the identification of patterns on the incidence of TBI.

### Quality of Studies

The articles ranged in STROBE scores from 9 to 22, with an average of 19 and median of 20. All studies had moderate (*n* = 3) or good (*n* = 26) methodological quality. The most common deficiency among the included studies was the failure to describe efforts taken to address potential sources of bias (*n* = 9). Articles with methods described elsewhere corresponded to a lower score (*n* = 2).

### Injury Characteristics

#### Overview

A total of 242,731 baseball- and softball-related TBIs [sum of column “#TBI(%)” in Table 2] were captured across the twenty-nine studies included in this review. These do not represent the true number of TBIs in baseball and softball, but clearly identify TBI as a concern to baseball and softball players. For youth (under eighteen) baseball, TBIs accounted for 5.8% of all baseball-related injuries (25). Although a consistent definition of TBI was not found among the included studies, concussion was the most commonly identified outcome of interest (Table 3). Further, TBI rates varied according to sex (22), level of play (19, 20) (high school, collegiate, major, and minor leagues) and whether it was a game or practice (18, 28). The average injury rate (Table 4) per 1,000 athletic exposures (AE) was 0.13 (range 0.03–0.46) (20, 33). The most common mechanisms of injury (Table 5) included being struck (by a ball or bat) (17) and colliding with another player (31). The distributions across these common mechanisms, however, varied according to age (17), gender (27), and level of play (20, 31). Finally, an increase in baseball- and softball-related TBIs was observed over time (26).

#### Changing Epidemiology

Despite the inclusion of many longitudinal studies in our review, few examined the changing epidemiological trends of baseball-and softball-related TBI. Nevertheless, the risk of concussion in high school boys’ baseball and girls’ softball increased between academic years 1997/1998 and 2011/2012 (26, 33). In emergency rooms however, the relative frequency of baseball-related brain injuries observed in youth ages 5–19 decreased from 1991 to 2008 (17). Importantly, the estimated number of total baseball related injuries in youth (i.e., sprains, soft tissue injuries, lacerations etc.) seen in emergency rooms also decreased between 1994 and 2006 (25).

Several studies using the NCAA database examined concussion rates in women’s softball and men’s baseball. Total concussion rates per 1,000 AE in collegiate women’s softball were 0.14 (1998–2004) (18), 0.19 (2005–2006) (19), 0.23 (2004–2009) (16), and 0.26 (2011–2015) (23), suggesting an increase in concussion rate over time. In collegiate men’s baseball however, total concussion rates per 1,000 AE were 0.07 (1998–2004) (18), 0.09 (2005–2006) (19), 0.12 (2004–2009) (16), and 0.09 (2011–2015) (23).

Finally, it is important to note the growing research on TBI in recent years. Of the 29 included studies, 2 were published before 2000, 4 were published 2000–2005, 9 were published between the years 2006–2010, and 14 studies were published after 2010 (Table 2). This growing trend in TBI research may correspond to an increase in awareness and education surrounding TBI, and consequently influence the reported rate and incidence of injury.

#### Mechanisms of Injury

Mechanisms of injury were explored in thirteen studies (Table 5). Being struck by an implement (either ball or bat) was most the common mechanism of TBI among all age groups, and accounted for 75.4% (100,773/133,739) of reported TBIs in these studies (2–5, 13, 14, 17, 19, 20, 27, 28, 31, 37).

Eight studies identified the ball as the major cause of baseball- and softball-related TBI (2, 13, 14, 17, 19, 20, 28, 37). The percentage of TBIs due to being struck by the ball ranged from 17.5% (31) to 75% (13), with the majority of injuries resulting from pitched (48.5%) and batted (37.4%) balls (20). A study by Birchak et al. (13) found that being hit by a ball was 2.81 times more likely to affect the head than any other mechanism of injury. Being hit by a pitched ball resulted in a greater proportion of TBI in men’s baseball (26.1%) compared to women’s softball (5.2%) (27).

Collisions were another common mechanism of TBI (2, 31). Among high school and collegiate athletes, collision accounted for 55.0% (31) of TBIs in one study and 39% (2) in another. Furthermore, 29.6% of TBIs in US major and minor baseball leagues were due to collisions with another athlete (20).

For children ages 5–9, the bat was again identified as the main culprit of TBI. Being hit by a bat accounted to 53.8% of TBIs in boys and 60.9% in girls (17). Among 5–9 year-olds seen in the ED, being hit by bat was the most common mechanism of TBI for both male (53.8%) and female (60.9%) baseball players, often due to being too close to the batter (males: 26.1%, females: 28.3%) (17). For children ages 10–19 year-olds however, the most common mechanism was being hit by the baseball for both male (58.0%) and female (70.1%) players (17).

#### Activity and Field Location

There were statistically significant differences in the number of TBIs based on the type of baseball activity players were engaged in at the time of injury (20). In the major and minor leagues, 49.2% of TBIs occurred at the home plate (20). In collegiate women’s softball, the majority of TBIs (32%) occurred to the batter (28) while the majority of TBIs (30.3%) occurred to the middle infielder in collegiate men’s baseball (18). Among high school athletes, the proportion of concussion to the total number of baseball-related injuries did not differ dramatically according to player position (range 2.9–4.6%) (3). However, the majority of TBIs occurred to the batter in men’s baseball (50.6%) and to the catcher in women’s softball (29.7%) (19).

#### Use of Protective Equipment

The use of protective equipment during TBIs was only reported in five studies (Table 5) (2–5, 17). While helmets are mandatory in all formal baseball leagues (4, 5), recreational baseball players rarely used helmets (2, 17). Studies involving high school and collegiate teams, generally found poor compliance for the use of helmets, or any protective equipment (2, 3). Further, among the baseball-related TBI cases seen in the ED, only 6.7% reported the use of a helmet, 12.7% had no protection of any sort, and 74.1% did not specify whether protective equipment was worn (17).

Upon examination of catastrophic head injuries in high school and college baseball, 0/26 players wore a helmet at the time of injury, 9/26 players suffered a skull fracture, 3/26 players sustained a subdural hematoma, and 5/26 injuries were fatal (2). While helmet hit-by-pitch incidents are a leading cause of concussion in MLB, a retrospective case-control study found that 9/18 (50%) of helmet hit-by-pitch resulted in a concussion diagnosis (4). In this study, the average pitch velocity that resulted in a concussion was 91.6 mph (compared to 90.8 mph for pitches that did not result in a concussion upon impact), no players observed loss of consciousness and the average days missed for concussed players was 14.2 (4).

#### High School vs. Collegiate Athletes

Eight studies, spanning the academic years 1995–2012, examined TBI of high school baseball and softball players (Table 2) (3, 19, 26, 27, 31–33, 36). Injury rates per 1,000 AE (Table 4) ranged from 0.03 (33) to 0.16 (27). Unanimously, the studies concluded that high school females were at a higher risk of TBIs than males, with average injury rates of 0.10 and 0.04 per 1,000 AE, respectively (19, 26, 27, 31, 33, 36).

Collegiate baseball and softball was the focus of eight studies, and together, they covered the years 1988–2015 (16, 18, 19, 22, 23, 28, 35, 38). Injury rates per 1,000 AE ranges from 0.03 in men’s baseball practices (19), to 0.42 in women’s softball competitions (23). As with high schools, these studies consistently concluded that females were at higher risk of TBIs than males, with total average injury rates of 0.08 and 0.18 per 1,000 AE, respectively (16, 19, 22, 23, 31).

Two additional studies compared TBIs in high schools and colleges across the US (2, 29). Overall, injury rates were generally greater for collegiate athletes compared to high school athletes (2, 19, 29).

#### Sex Differences

Fourteen studies examined gender differences in TBI with specific comparisons between baseball (males) and softball (females) (Table 4) (16, 18, 19, 22, 23, 26–29, 31–33, 36, 38). TBI rates (per 1,000 AE) were on average 2.04 times greater in females playing softball compared to males playing baseball (16, 18, 19, 22, 23, 26–28, 31, 33, 36, 38). Other research, that did not segregate males to baseball and females to softball (and included both sexes in baseball or softball) reinforced these findings illustrating females had a higher risk of TBI than males (13, 17). TBI injury rates were not available for youth under the age of 19 playing outside of an organized setting (i.e., outside of high school and little leagues).

#### Practice vs. Game Setting

In the eight studies that compared TBIs in practices and games, injury rates (per 1,000 AE) were found to be higher in a game setting (Table 4) (16, 18, 19, 23, 27, 28, 31, 38). Overall, the game-practice rate ratio was 4.17. The average rate ratio was greater for baseball (5.65; range 2.67–11.00) (19, 27) than for softball (2.58; range 0.44–5.29) (19).

The vast majority, 90.2% (*n* = 277) of TBI among professional baseball players in the major and minor leagues occurred in games, 1.6% (*n* = 5) in practice, 4.5% (*n* = 11) in spring training, and the remaining 3.6% (*n* = 11) from off-field, non-baseball injuries (20).

Among high school athletes, the proportion of concussions relative to all baseball and softball injuries were higher in competition than in practice for men’s baseball (2.5% and 1.8%, respectively), but lower in competition than in practice for women’s softball (0.04 and 8.9%, respectively) (32). Among collegiate athletes however, concussion represented a greater proportion of total injuries in competition than in practice for both baseball (4.2% of game injuries and 2.9% of practice injuries) and softball (6.4% of game injuries and 4.1% of practice injuries) (38).

#### Post Injury

The severity of baseball- and softball-related TBIs vary considerably, from mild and returning to the field on the same day as injury, to immediate death (Table 6) (2, 36). In a descriptive epidemiologic study examining 100 US high schools and 180 US colleges, Gessel et al. (19) found that 64.2% (1,279/1,992) of baseball and 68.8% (2,449/3,560) of softball players had symptoms resolved within 6 days following injury.

Seven other studies examined the recovering status of athletes and time for symptom resolution, covering a total of 227 TBIs (Table 6) (2, 4, 29–31, 34, 35). Generally, injuries were mild, with 28.6% (65/227) players making a full recovery and symptoms resolving within one week (2, 29, 31, 34). Moderate injuries, where symptoms resolved within 7–30 days following injury, accounted for 22.5% (51/227) of TBI (31, 34). 72 athletes (31.7%) had symptoms lasting less than 30 days, with exact time of symptom resolution unspecified (4, 30, 35). Injuries that required surgical procedures, resulted in disability or persisting symptoms were considered severe, and accounted for 14.1% (32/227) of TBI (2, 29, 34, 35). In total, eight athletes (3.5%) died following a TBI (2, 29). Of these deaths, three were fielders hit by a batted ball, one was a fielder colliding with another athlete, and one was a batter hit by a pitched ball (2). The mechanisms of the remaining three fatalities are unspecified (29). Interestingly, more recent studies published in 2015 and 2016 did not report any fatalities (34, 35).

Among MLB players, 53% of concussions resulted in the player being placed on disabled leave (34). Compared to players on bereavement/paternity leave, concussed players that returned to play after they had recovered have significantly worse batting performance in nearly all metrics including: batting average, on-base percentage, slugging percentage, and home run percentage (34). Such differences, however, resolve 4–6 weeks after returning to play (34).

A study by Boden et al. (2) that focused on 26 catastrophic head injuries found that 5/26 (21.7%) players died from severe cerebral bleeding immediately after a head injury and 8/26 (30.8%) required one or more surgical procedures. Recovery analysis revealed that 11/26 (42.3%) of athletes suffered from ongoing neurological deficiencies/symptoms, and only 4/26 (15.4%) returned to competitive play (2). Further, Lawson et al. (25) noted that 5.8% of all baseball injuries that were brought to the ED were diagnosed with concussion/closed head injury, and 17.7% of the injuries that required hospital admission after ED visit were due to concussions/closed head injuries.

#### Returning to Play

Two concussion RTP guidelines were assessed for compliance rates among concussed high school baseball players; The 1997 American Academy of Neurology (AAN) (39) and the 2005 Prague (40) RTP guidelines (Table 6). This analysis revealed a 100% compliance rate with the AAN guidelines for athletes diagnosed with Grade I concussions, however, these rates dropped for both Grades II and III (36). Indeed, 56% of males and 32% of females with diagnosed with a Grade II concussion were non-compliant with the AAN RTP guidelines (36). Under the Prague RTP guidelines however, overall non-compliance rates dropped to 12% for both boys baseball and girls softball (36).

## Discussion

This systematic review of research studies examining the relationship between baseball and softball and TBIs identified only twenty-nine articles on the topic. In part, this is because there were no baseball and softball injury data available until the formation of the National Center for Catastrophic Sports Injury Research (NCCSIR) in 1982 (29). The identified studies in our review found variance in the reported proportion of TBIs per injury case. This is not surprising when considering that there was no consistent definition of TBI among the included studies. To date, the vast majority of the current literature studied baseball- and softball-related TBIs among other baseball and softball injuries (i.e., musculoskeletal injuries) and/or other sports (i.e., baseball/softball included with the study of other sports such as hockey, football, soccer etc.), with only one article investigating solely TBIs in baseball players (20).

Several trends were observed. First, while being struck by a bat was the most common mechanism of injury for younger players (17), being struck by a ball was the most common mechanism for older athletes (adolescent and beyond) (2, 13, 14, 17–20). Second, batters and catchers were at the highest risk of TBIs among all positions of play (18–20), with the majority of TBIs occurring at the home plate (19, 20). These positions also experience the highest overall injury rate in baseball, thus the proportion of TBIs to total injuries does not differ dramatically across the different positions (3). Third, the current review suggests the rate of TBI in baseball and softball to be 0.03–0.46 per 1,000 AE, with variations attributed to gender, setting, and levels of play (20, 33). Baseball and softball were found to have the lowest incidence of concussion (22, 23, 26), when reported alongside fifteen other sports. For example, the rate of TBI has been reported to be 0.75 per 1,000 AE in American football and 0.74 per 1,000 AE in ice hockey (23). Despite the seemingly low injury rate, concussions are among the top ten time-loss injuries in professional baseball (41), can be career threatening for athletes (42) and have potentially catastrophic results, including immediate death (2). TBIs accounted for 63.4% (26/41) of all catastrophic baseball injuries reported to the NCCSIR between 1982 and 2002 (2). It is important to note, however, that more recent studies did not report fatalities as a common outcome of baseball- and softball-related TBI (34, 35). This may be a result of increased awareness and education surrounding sports-related TBI, improved equipment, and rule enhancement and/or rule changes to enhance the safety of the athletes. While fatalities seem to have decreased, the rate and incidence of TBI continues to increase in baseball and softball (16, 18, 19, 23). As such, the findings of this review support the need for comprehensive exploration and analysis of TBIs in baseball and softball.

Our review highlights the importance of TBIs in baseball and softball. It is imperative for both medical personal, individual athletes, and others involved in the game and its organization and implementation to exercise greater caution and develop better awareness regarding the potential severity of TBIs, regardless of how minor the injury may first appear.

Collegiate athletes were more commonly injured than high school athletes, and the sustained TBIs were more prevalent during competition than practice (16, 19, 23, 27, 28, 31, 32, 38). This finding is consistent with the reports of eight other sports, including football, soccer, volleyball, basketball, and wrestling (19). The disparity between competition and practice may be due to the increased likelihood of contact with other players or bases during games (28). This is supported by the finding that collisions are among the most common mechanism of injury among players (2, 20, 31). In addition, a game setting fosters elevated competitiveness and aggression which has been found to put players at a higher risk of TBIs in other sports such as hockey (43, 44). It is reasonable to assume this finding translates to baseball and softball as well. An alternative explanation for the higher prevalence of TBIs found in games may be a result of the closer monitoring of athletes, resulting in more injuries being reported to health care professionals.

Physical differences related to athlete strength may contribute to why TBIs are more prominent among collegiate players compared to the high school population. Collegiate players may be colliding with greater forces or pitching at a greater velocity. However, there are conflicting views regarding the extent to which this may impact the proportion of concussion in total baseball and softball injuries between these two groups of players. One argument states that the greater intensity of collegiate level baseball and softball and the athlete’s enhanced speed and strength explains why a higher rate of concussion might be expected from collegiate athletes (15, 19). Conversely, the potential for more playing time, lower skill level, and lower quality of equipment of high school teams allows others to argue that higher proportions of concussion should be observed by high school players (45). The truth likely lies somewhere in between these opposing views and that the real difference in concussion proportions between high school and collegiate athletes are small.

This review found that while overall, based on reported cases, more males sustained TBIs than females, but females have a higher injury rate (16, 18, 19, 22, 23, 26, 27, 31, 32, 38). This is an interesting finding as female players are typically associated with softball where the balls are larger, but are less dense and have a softer core. Experimental studies suggest that softer balls reduce the impact response of head and chest models and should therefore be safer (46). Higher rates of concussion for females were also reported in other sports such as soccer and basketball (26), suggesting that cofounding factors may have contributed to the higher TBI prevalence observed in women’s softball. Females may be more likely to report an injury than males. Only 47% of high school football players suffering from concussion symptoms chose to report their injury (47), because they did not think their injuries were severe enough to warrant medical attention and/or feared that they would be removed from the game. In addition, North American societies have traditionally been more protective of female athletes, which may cause coaches, parents, and athletic trainers to treat TBIs in females more seriously than in males (19, 48). Alternatively, it has been suggested that there may be neuropsychological differences in the susceptibility to TBI and concussion symptoms among sexes (38, 49).

The findings of our review illustrate an increase in the rate of baseball- and softball-related TBIs over the past decade (12, 26, 28). This is consistent with overall increase in concussion rates observed across all sports (including football, hockey, basketball, etc.) (22, 26). The increase in concussion rates may reflect a true increase in the risk of sports-related TBIs, but it may also be attributed, at least in part, to improvements in the identification, monitoring, and reporting of TBIs (22, 26). While emergency room visits for concussions are increasing, participation in organized team sports is decreasing (12), thus demonstrating the need of for effective injury prevention programs.

## Framework for Prevention of Baseball- and Softball-Related TBI

The findings of our review illustrate a need to prevent TBI in baseball and softball. Although prevention strategies may differ for different ages and levels of competition, these findings support the use of preventive measures to limit injury prevalence among these athletes. Further, despite the differences in softball and baseball, the mechanisms of injury are similar and therefore have similar avenues of injury prevention. A simple Haddon’s Matrix (1968) (50) was constructed to organize preventative measures (Table 7).

### Haddon’s Matrix for Prevention of TBI in Baseball and Softball

Developed by William Haddon Jr., the Haddon matrix is commonly used injury prevention research and intervention (50, 51). The grid has four columns representing different influencing factors (host, agent, physical environment, social environment) that contribute to the injury process. The grid also consists of three rows, each representing different phases of an injury (pre-injury, injury event, post-injury). The matrix combines public health concepts of primary, secondary, and tertiary prevention with the concepts of host-agent-environment as target of change for delivering public heath interventions (51). Each cell of the matrix represents a distinct locus for identifying intervention strategies for addressing a public health concern (51).

Applying the Haddon matrix to the threat of TBI in baseball and softball facilitates public health agencies and stakeholders to conceptualize, predict, prevent, and mitigate injury (Table 7). The host column represents the person or persons at risk of injury. These factors include the age, sex, and strength of athletes, as well as an athlete’s knowledge of TBI and compliance to return-to-play guidelines. The agents of injury include the ball, the bat, and a second player (*via* collision). Physical environment refers to the setting of where the injury occurs. These factors include the condition of the field, time of year, use of personal equipment, as well as access to appropriate medical care. Sociocultural and legal norms of a community constitute the social environment. Such factors include the public perception of helmet use, regulation and enforcement of protective gear use, and community response to TBI. Economic factors include the cost of protective gear, cost of medical care as well as insurance rates. Haddon’s matrix provides a framework for understanding of the origins of injury and for identifying multiple countermeasures to address TBI in baseball and softball.

### Interventions for Injury Prevention

In terms of specific interventions, we propose the following:

(A) *Educate athletes, parents, and coaches about TBI; its symptoms and importance in preventing and reporting injuries*. TBI in baseball and softball can have serious consequences including immediate death and permanent neurological effects (2). Thus, it is important for all participants to recognize the risks and potential threats of TBIs, and the necessity of reporting an injury regardless of how minor the injury appears to be upon first impressions. TBI education is a potential solution to reduce the reluctance of athletes to report injuries. Concussion educated soccer players, for example, are more likely to report a suspected concussion than uneducated players, thus reducing their risk for further injury (52). Education can also be formulated around taking safety precautions to prevent TBIs. For instance, young players are especially prone to being hit by a bat due to standing too close to the batter; a practice that can easily be prevented with education around creating safe distances from the batter (17). Education needs to be directed broadly to players, coaches, trainers, parents, officials, and those responsible for the organization of baseball and softball like school officials and league directors.
(B) *Enforcement of rules, laws, and the use of helmets*. The Zackery Lysted Law (ZLL) (2009), named after a young athlete who is permanently disabled after sustaining a concussion due to prematurely returning to a play football, requires “school districts and non-profit organizations using school facilities to adopt policies for the management of concussion and head injury in youth sports” (53). Our study found that up to 55.6% of concussed baseball and softball athletes were non-compliant with RTP guidelines (36). This player non-compliance likely reflects a broader culture of non-compliance within the sports organizations and people such as team officials, coaches and trainers, parents, peers, and sponsors that create a permissive environment that does not discourse this sort of behavior amongst players. Since the numbers are reported from schools with a NATA affiliated certified athletic trainer (36), we suspect non-compliant rates to be even higher in schools without the guidance of a certified trainer. This is of major concern as pre-mature RTP has been shown to increase the risk of re-injury (54), result in more severe post-concussive symptoms (55) and decrease an athlete’s reaction time (56).
  The most recent guidelines on concussion and sport developed in 2013 (57) require a thorough evaluation of their effectiveness. However, they are likely to be more comprehensive and have the potential to be more effective at reducing the burden of TBI and minimizing attrition from sport than earlier guidelines.
  The effectiveness of helmets as a means of protection against TBI has raised some controversy. For instance, a 2000 systematic review examining the effectiveness of helmets in preventing head and brain injuries in cyclists found that helmets reduce the risk of TBI by up to 88% (58). A more recent review, however, concludes that helmet use does not result in better clinical outcome or protection against concussion (59). Controversy surrounding helmet use against concussion may be a result of the lack of a standardized definition of concussion and/or a lack of standardization in examining helmet efficacy (59). Nevertheless, there is significant literature supporting the effectiveness of helmets in protecting against moderate to severe TBI resulting in disability or death (2, 59). In particular, helmets provide clear protection against open skull fractures, intracranial hemorrhage, and penetrating head injuries (2, 59–61). As such, we believe that it is important to enforced and encourage the use of helmets among all baseball and softball players.
(C) *Engineering of new helmet, bat, and ball designs to minimize the force of an impact*. The protective efficacy of helmets may vary by type and design (5, 59). As such, clinicians, scientists, and engineers must work together to create helmets that are effective in protecting athletes against TBI. The average pitch velocity of 86.2 mph among MLB players is considerably higher than the current standard test speed of 70 mph for faceguards (4, 5). In addition, the dominance of aluminum bats over wooden bats in modern day baseball and softball allows players to swing faster due to their lightweight. With the major culprit of TBIs in baseball and softball being the ball itself, advances in ball design that increase the speed of balls should be taken cautiously to minimize injury risks. Recommendations for potential face guards on batting helmets should also be considered in reducing injury risk (62).
(D) *Economic incentives and disincentives; fines, insurance rates, price of equipment, and medical care*. Socioeconomic status, availability of financial resources and level of insurance coverage available has shown to influence outcomes after TBI (63). Although not explored in the literature we reviewed, financial factors and economic limitations play a role in preventive efforts (63). For instance, the cost of medical care may be limiting factor to the number of athletes seeking professional help after an injury. The price of helmets and other protective gear may also contribute to the poor rates of helmet use, particularly in school districts with lower average income. Given that for every dollar that is spent on bicycle helmets, over $30 is saved in medical costs (64), this would be a good investment for all leagues and schools. Providing incentives and disincentives in the form of insurance policies for those complying with safety initiatives and lower rates of TBI can also potentially have beneficial effects.
(E) *Surveillance and evaluation*. Timely and accurate injury surveillance systems play a major factor in our understanding of TBI rates and outcomes. They are also critical in studying the effectiveness of preventive strategies such as the ZLL. While recent examination of ZLL shows promise in reducing some sports-related TBIs (such as cycling) among youth, future empirical research is required to determine whether such legislations are effective in preventing TBIs in other sports (65). Efficient surveillance systems require investments by organized groups in sports to develop and to maintain. A willingness to act on the data that arises from such systems is also crucial in creating effective methods of injury prevention.

## Limitations

### Study Limitations

There were several limitations to this systematic review. Differences in the definition of TBI across the twenty-nine studies made the comparison between results challenging. Variability in the reporting of TBI adds further challenges, as the methods used to diagnose TBI across the studies remain unclear. In addition, although the included studies were subjected to quality assessment, we could not resolve any intrinsic problems with the design of the primary studies. While the majority of the studies attempted to use samples collected from a well-established database (i.e., NEISS, CHIRPP, RIO), all data collection methods have limits. For example, the database RIO is based on self-reporting surveys, is highly subjective and prone to errors in injury diagnosis. Furthermore, most of the studies were limited to samples obtained from hospital emergency rooms or schools with a NATA affiliated certified athletic trainer, thus potentially underestimating the total number of injuries, especially mild ones. In spite of these limitations, this review provides the first systematic analysis of the epidemiology of TBI in baseball and softball, important avenues for prevention, and outlines current gaps in the literature.

### Gaps in Current Knowledge and Future Research

We identified several gaps in the current literature. First, no data were available for TBIs associated with T-ball. Second, only twelve studies reported the post injury outcomes and among those, the differences in the selected participants (i.e., ED visits, fatal head injuries) produced huge variations among presented results. As such, the current literature provides little information on the severity and outcomes of baseball- and softball-related TBIs. Third, although twenty-nine studies were included, only thirteen explored details of injury (i.e., mechanism). Thus, it is important to note that very few studies focused specifically on baseball- and softball-related TBIs, and there is currently little available information on the specific nature of TBIs acquired during baseball play. For example, the risk of TBI among informal play (i.e., on the playground, at home) remains largely unknown. Furthermore, of the studies that reported mechanisms of injury (*n* = 13), all reported on the biomechanical loading mechanism (i.e., hit by ball, hit by bat, player collision, sliding). No studies reported on the biochemical secondary injury mechanism (pathophysiological damage leading to concussion, contusion, or hemorrhage). Given that fourteen of fifteen studies in softball reported on outcomes in females alone, there is considerable room for more research on males in softball. Geographic location also remains a major limitation as twenty-seven of the twenty-nine included studies were based on populations in the United States. Future work should determine if systematic differences in how the game is played in different regions like the Caribbean countries or Japan have any effect on rates and causes of TBI. Similarly, the relationship between of social and economic factors and the rates of TBIs are largely unexplored. Moreover, more research should be directed to younger ages, t-ball related TBIs, and informal play. Finally, the results of our STROBE analysis demonstrate a need for authors to describe efforts taken to address potential sources of bias when reporting TBI in baseball and softball. Such information is not only necessary to direct future research but also important for the general public to take adequate measures of injury prevention.

## Conclusion

This systematic review found twenty-nine articles that detailed TBIs in baseball and softball covering participants of all ages. The most common mechanisms were struck by implement and collision with another player or object. The most explored outcome was concussion. Resistance to helmet use and non-compliance with RTP guidelines remains a lingering concern, particularly among high school and collegiate athletes. There is a need for preventive measures to focus on education for all ages and for all players, parents, coaches, and league officials. All stakeholders should be aware of the ZLL and advances in ball, bat, and helmet design. Current surveillance programs should be evaluated pertaining to their effectiveness and risk of bias. Further research on the quality of different helmets, severity of injuries, and risks associated with playing outside a formal setting is also required. Such information would not only provide a greater understanding to baseball- and softball-related TBIs but also aid in the development of prevention and management modules.

## Author Contributions

MC contributed to the conception and design of the work, while AZ contributed to the acquisition, analysis, and interpretation of data for the work. AZ drafted the work, while MC revised the work critically for important intellectual content. Both MC and AZ approve the final version to be published and agree to be accountable for all aspects of the work in ensuring that questions related to the accuracy or integrity of any part of the work are appropriately investigated and resolved.

## Conflict of Interest Statement

The authors declare that the research was conducted in the absence of any commercial or financial relationships that could be construed as a potential conflict of interest. The reviewer, JL, and handling editor declared their shared affiliation.
